# Effects of Hybrid Copper Oxide-Silver and Titanium Dioxide-Silver Nanocoatings on the Surface Roughness and Friction of Stainless Steel Orthodontic Brackets: A Comparative In Vitro Study

**DOI:** 10.7759/cureus.118793

**Published:** 2026-10-29

**Authors:** Devireddy Alekhya, Panaka Padma Prathyusha, Greeshma M Harini, Perala Johnson, Uday Kumar Digumarthi, Ganugapanta Vivek Reddy, Gowri Sankar Singaraju, Prasad Mandava

**Affiliations:** 1 Orthodontics and Dentofacial Orthopedics, Narayana Dental College and Hospital, Nellore, IND; 2 Orthodontics and Dentofacial Orthopedics, Care Dental College, Guntur, IND

**Keywords:** atomic force microscopy, copper oxide, frictional resistance, nanoparticle coating, orthodontic brackets, silver nanoparticles, surface roughness, titanium dioxide

## Abstract

Introduction

Nanoparticle coatings on orthodontic brackets have antibacterial potential but may alter surface roughness and friction at the bracket-archwire interface. This study compared the surface roughness and static frictional resistance of stainless steel brackets coated with silver-impregnated copper oxide (CuO+Ag) or silver-impregnated titanium dioxide (TiO₂+Ag) nanoparticles with those of uncoated brackets.

Materials and methods

Thirty stainless steel maxillary central incisor brackets (0.022-inch slot) were allocated into three groups (n = 10 each): uncoated control, CuO+Ag-coated, and TiO₂+Ag-coated. Arithmetic average roughness (Ra), root mean square roughness (Rq), and maximum peak-to-valley height (Rz) were measured by atomic force microscopy as received (T0), after coating (T1), and after friction testing (T2). Static friction was measured using a universal testing machine with a 0.019 × 0.025-inch stainless steel archwire in artificial saliva. Data were analyzed using one-way or Welch analysis of variance with post hoc tests and paired t-tests (p < 0.05).

Results

Baseline roughness was comparable among the groups. At T1, CuO+Ag increased Ra, Rq, and Rz, whereas TiO₂+Ag increased Ra and Rq but decreased Rz (paired t-test, |t(9)| ≥ 10.82, p < 0.001). Static friction differed among the groups (F(2, 27) = 9.15, p < 0.001, η² = 0.404). CuO+Ag showed the lowest friction (36.31 ± 3.90 gf), significantly lower than the control (41.65 ± 4.77 gf; q = 3.82, p = 0.031) and TiO₂+Ag (44.65 ± 4.53 gf; q = 5.97, p < 0.001), whereas TiO₂+Ag did not differ from the control (q = 2.15, p = 0.298). Roughness increased in all groups after friction testing (t(9) ≥ 33.48, p < 0.001), with smaller increases in the coated groups.

Conclusions

Both nanocoatings altered bracket surface roughness. CuO+Ag significantly reduced static frictional resistance and appears promising for further investigation as an orthodontic bracket coating.

## Introduction

Fixed orthodontic appliances favor plaque accumulation and microbial colonization around brackets, contributing to white spot lesions during treatment [1,2]. Because preventive measures often depend on patient compliance, surface modification of orthodontic appliances has received increasing attention as a strategy to reduce bacterial adhesion and biofilm formation [3].

Nanoparticles are attractive for this purpose because of their distinctive physicochemical and antimicrobial properties [4]. Silver (Ag), copper oxide (CuO), and titanium dioxide (TiO₂) nanoparticles have been incorporated into orthodontic materials, with recent studies showing reduced cariogenic bacterial counts on coated brackets [5,6]. Systematic reviews support this antimicrobial potential, although clinical evidence remains limited [3,7].

Hybrid coatings may provide additional benefit. CuO/Ag nanocomposites have shown enhanced antibacterial activity [8], while TiO₂/Ag-coated brackets have demonstrated anti-adherent and antibacterial effects against *Streptococcus mutans* *(S. mutans) *and *Porphyromonas gingivalis *(*P. gingivalis*)* *[9]. Other hybrid coatings, including AgCuO, have also shown favorable antimicrobial effects [10,11].

However, coating the bracket slot may alter the surface over which the archwire slides. Surface roughness can influence frictional resistance, although this relationship is complex [12,13]. A systematic review suggested that nanoparticle coatings may reduce friction, but much of the available evidence concerns coated archwires rather than coated brackets [14]. Findings from coated brackets remain inconsistent: some Ag-, titanium oxide-, and Ag-chitosan coatings increased friction [15,16], CuO and TiO₂ coatings showed no significant difference from uncoated brackets [17], and other nanocoatings have either increased or reduced friction depending on coating composition and characteristics [18,19].

Therefore, the effect of hybrid nanoparticle coatings on bracket surface roughness and frictional behavior remains unclear. This in vitro study aimed to compare the surface roughness and static frictional resistance of stainless steel orthodontic brackets coated with Ag-impregnated copper oxide (CuO+Ag) or Ag-impregnated titanium dioxide (TiO₂+Ag) nanoparticles with those of uncoated brackets. Surface roughness was assessed before coating, after coating, and after friction testing. The null hypothesis was that no significant differences would exist among the three groups in surface roughness or static frictional resistance.

## Materials and methods

Study design

This in vitro experimental study was conducted in the Department of Orthodontics and Dentofacial Orthopedics, Narayana Dental College and Hospital, Nellore, India, in collaboration with Virtue Meta-Sol, Hyderabad, India. The study was reported with reference to recommendations for preclinical in vitro dental-material research [20]. The experimental workflow is shown in Figure 1.

**Figure 1:**
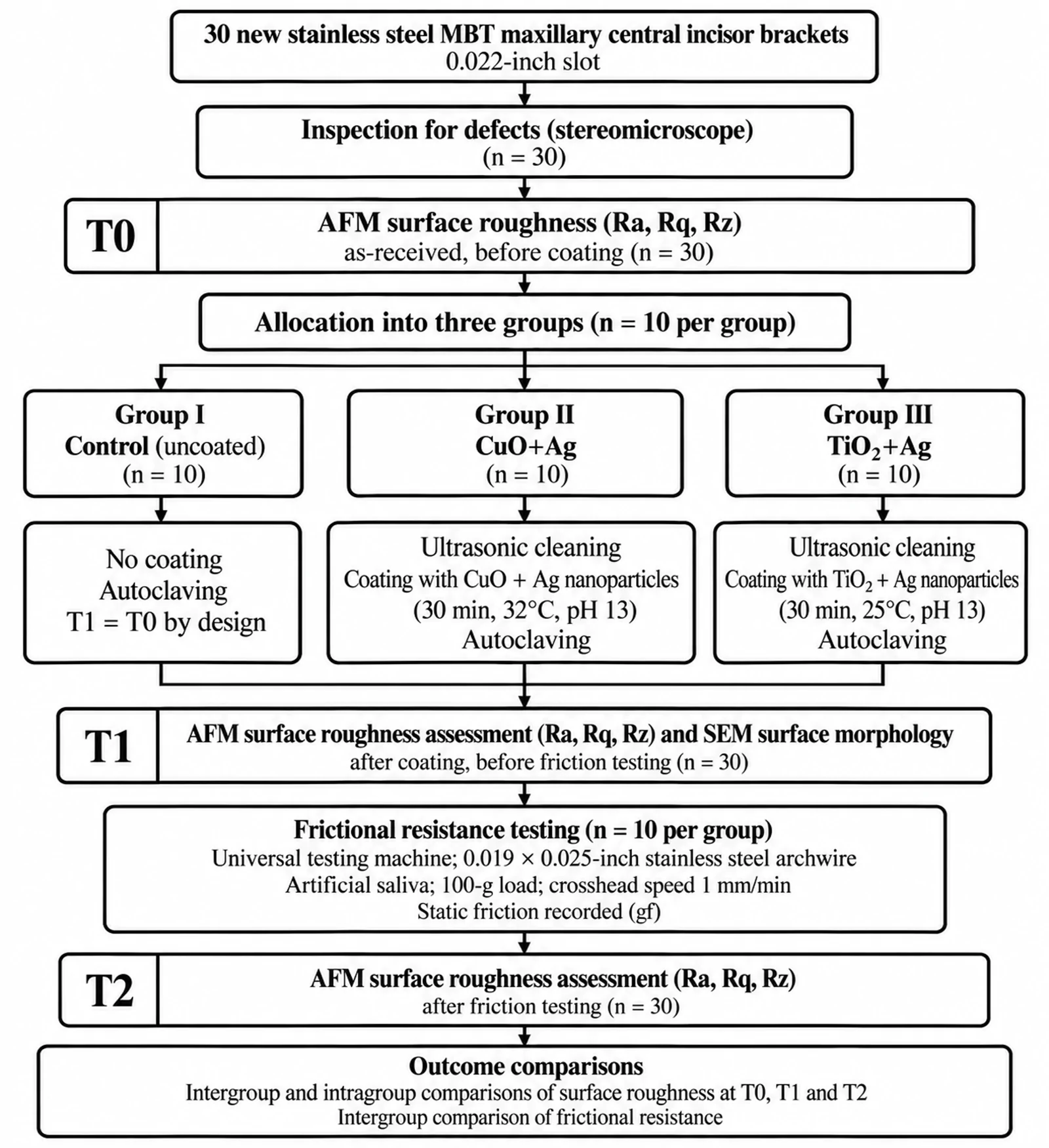
Study workflow and experimental design AFM: atomic force microscopy; SEM: scanning electron microscopy; Ra: arithmetic average roughness; Rq: root mean square roughness; Rz: maximum peak-to-valley height; CuO+Ag: silver-impregnated copper oxide; TiO₂+Ag: silver-impregnated titanium dioxide; gf: gram-force; T0: as-received condition before coating; T1: after coating and before friction testing; T2: after friction testing

Sample-size calculation

The sample size was calculated a priori using G*Power version 3.1.9.7 (Heinrich-Heine-Universität Düsseldorf, Düsseldorf, Germany) for a one-way analysis of variance (ANOVA) with three groups. The effect size was derived from frictional resistance values reported for uncoated and nanofilm-coated stainless steel brackets (0.55 ± 0.14 N, 0.77 ± 0.08 N, and 0.82 ± 0.11 N) [15], giving an effect size (f) of 1.04. With a significance level of 0.05 and a power of 90%, a minimum of six brackets per group was required. Ten brackets were included in each group, giving a final sample of 30 brackets.

Specimens and grouping

Thirty new stainless steel pre-adjusted edgewise maxillary central incisor brackets (Mini Diamond™, MBT prescription, 0.022-inch slot; Ormco, Mexico) were used. All brackets were inspected under a stereomicroscope, and brackets with visible surface defects, deformation, or manufacturing damage were excluded. Surface roughness of all 30 brackets was recorded in the as-received condition (T0) before allocation into three groups (n = 10 each). Group I consisted of uncoated stainless steel brackets and served as the control; Group II consisted of CuO+Ag-coated brackets, and Group III consisted of TiO₂+Ag-coated brackets. A 0.019 × 0.025-inch stainless steel straight archwire (Metro Orthodontics Pvt. Ltd., Hyderabad, India) was used for friction testing.

Surface roughness was evaluated at three time points: T0, the as-received condition before coating; T1, after coating and before friction testing; and T2, after friction testing. The same specimens were followed throughout the experimental sequence. Group I was not coated, and its T1 values were therefore identical to its T0 values by design.

Nanoparticle Coating Procedure

CuO, TiO₂, and Ag nanoparticles with a nominal particle size of 30-50 nm and purity of at least 99% were obtained from Nanospan India Private Limited, Hyderabad, India. The coatings were applied using a two-stage nanoparticle plating procedure comprising electrodeposition followed by electroless plating, based on the method described by Zhu et al. [21].

Before coating, the brackets were ultrasonically cleaned in ethanol and acetone for 15 minutes, washed in sodium hydroxide solution for 20 minutes, and rinsed twice in distilled water for 15 minutes each. The nanoparticles were ultrasonically cleaned in acetone and treated with 20 wt% hydrofluoric acid for five minutes.

In the first stage, the electrolyte was prepared from nanosilver solution, the respective metal oxide nanoparticles (CuO for Group II and TiO₂ for Group III), and sodium nitrate. A graphite electrode served as the cathode, and the brackets, held with crocodile clips, served as the anode, after which current was passed. The brackets were then rinsed and air-dried.

In the second stage, electroless plating was carried out for 30 minutes under mechanical agitation in a plating unit (Sun Motor Instruments, Hyderabad, India), at 32°C for CuO+Ag and 25°C for TiO₂+Ag. The pH of the solution was maintained at 13 using 20 wt% sodium hydroxide. The coated brackets were rinsed and air-dried, and all brackets, including the uncoated controls, were autoclaved before testing.

Frictional-Resistance Testing

Static frictional resistance was measured using a universal testing machine (FIE, India) fitted with a computerized data-acquisition system (Minitech Series 9000; DAK System Inc., Mumbai, India). A customized vertically mounted Plexiglass jig based on the experimental design described by Tidy was used [22].

A 16 × 16-mm window was prepared in the Plexiglass jig. Four brackets were bonded to the jig with cyanoacrylate adhesive (Fevikwik; Pidilite Industries, Mumbai, India) at 8-mm intervals, two on either side of the window, and a fifth movable test bracket was positioned within the window. All brackets were oriented identically. The archwire was ligated with elastomeric modules (Libral Traders, New Delhi, India), and the module on the test bracket was slackened to permit free sliding (Figure 2).

**Figure 2:**
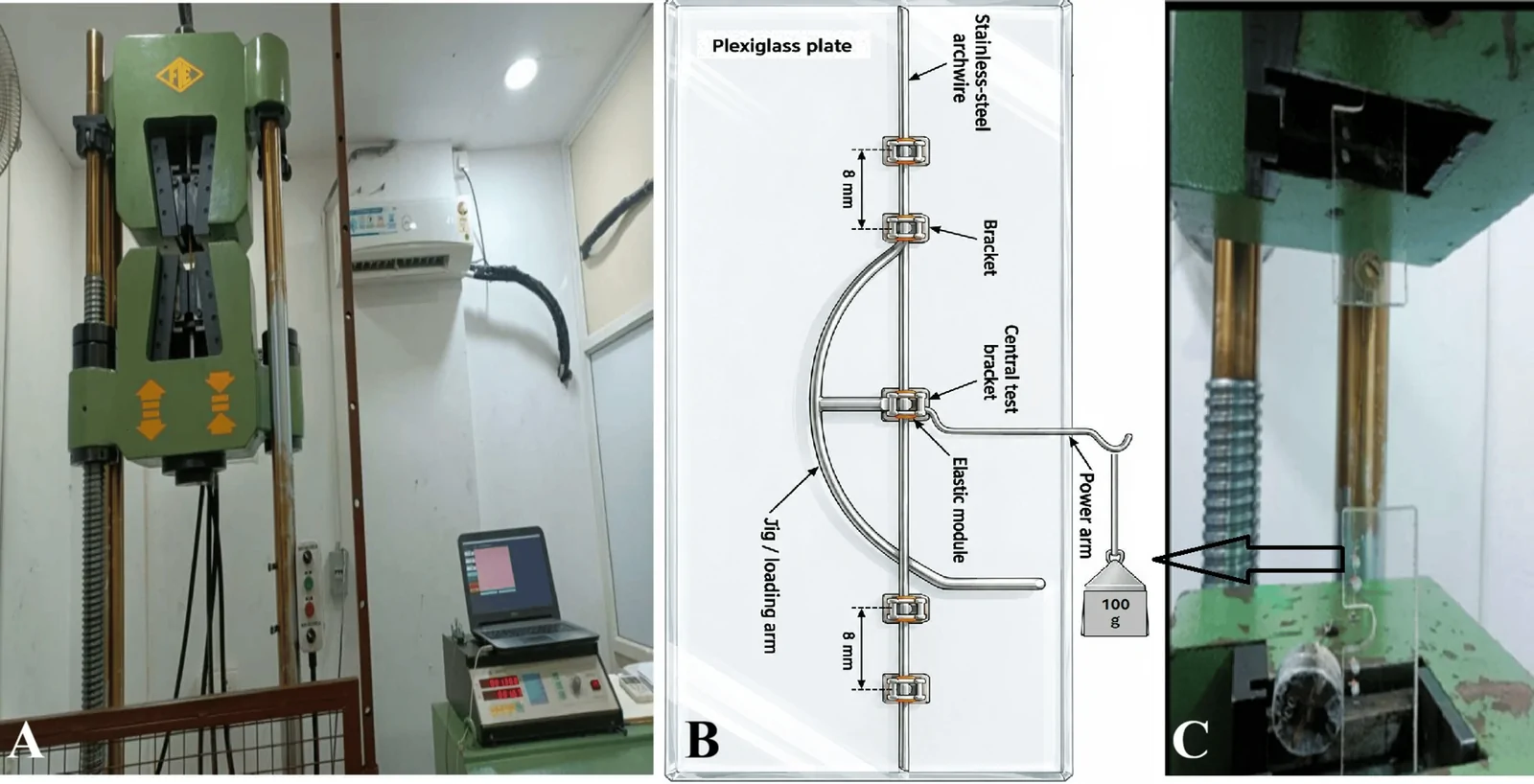
Experimental setup for frictional resistance testing (A) Universal testing machine (FIE, India) fitted with a computerized data-acquisition system (Minitech Series 9000; DAK System Inc., Mumbai, India). (B) Schematic representation of the customized Plexiglass jig showing four fixed brackets positioned at 8-mm intervals, two on either side of the central movable test bracket; a 0.019 × 0.025-inch stainless steel archwire was ligated with elastomeric modules, and a 10-mm power arm attached to the test bracket carried a 100-g load. (C) Bracket-wire assembly mounted on the universal testing machine during friction testing; the arrow indicates the power arm with the suspended load

The bracket-archwire assembly was immersed in Xerostat artificial saliva (Jiana Life Sciences, Mumbai, India) for one hour before testing. The test bracket was fitted with a 10-mm power arm carrying a 100-g load, and the bracket-wire angulation was maintained at 0°. A preliminary unloaded run was performed to ensure that the bracket was not binding on the archwire.

The archwire was drawn through the bracket assembly at a crosshead speed of 1 mm/min over a distance of at least 4 mm, and the force was recorded digitally using a 100-kg load cell. The first peak was recorded as the static frictional resistance and expressed in gram-force (gf; 1 gf = 0.0098 N). The same mounting jig was used throughout the study. A new archwire segment and new elastomeric modules were used for each test bracket, whereas the fixed brackets and the jig remained unchanged throughout the study.

Surface Characterization

Surface morphology was examined qualitatively using a scanning electron microscope (JCM-6000PLUS; JEOL Ltd., Tokyo, Japan) operated at 15 kV. Representative images were obtained at ×20 and ×200 magnification to characterize surface morphology and coating distribution. Coating thickness was not quantitatively measured (Figure 3).

**Figure 3:**
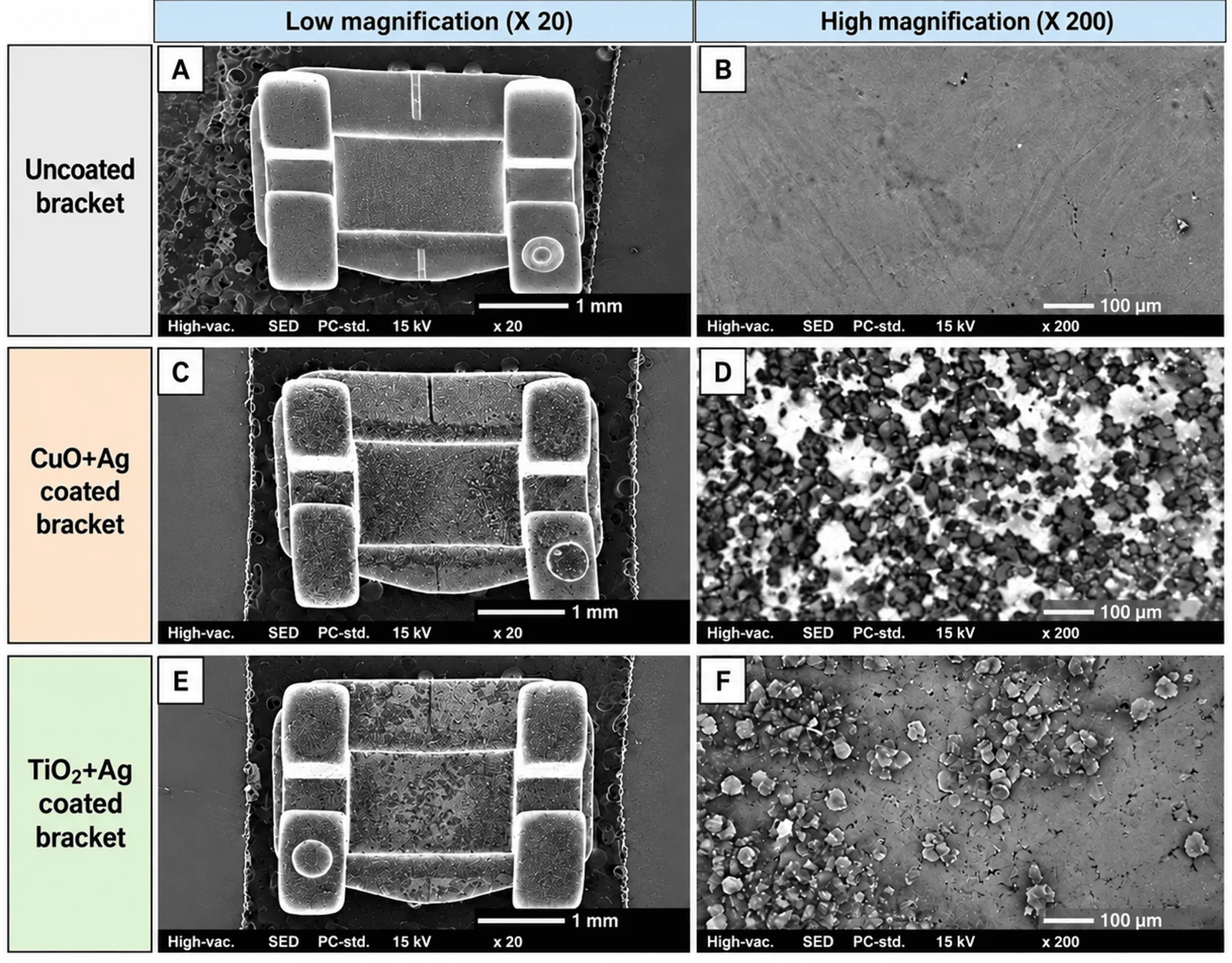
Representative scanning electron microscopy images of stainless steel orthodontic brackets CuO+Ag: silver-impregnated copper oxide; TiO₂+Ag: silver-impregnated titanium dioxide (A, B) Group I, uncoated control; (C, D) Group II, CuO+Ag-coated bracket; (E, F) Group III, TiO₂+Ag-coated bracket. The left column (×20; scale bar 1 mm) shows the overall bracket surface, and the right column (×200; scale bar 100 µm) shows the surface morphology at higher magnification. CuO+Ag-coated brackets showed dense, irregular particulate deposits, whereas TiO₂+Ag-coated brackets showed a comparatively more uniform particulate distribution

Surface roughness of the central portion of the bracket slot was assessed using an atomic force microscope (NX10; Park Systems, Suwon, Korea) with a scanner range of 100 × 100 × 5 µm in the x, y, and z directions. Images were acquired at a scan rate of 0.5 Hz and a resolution of 256 × 256 pixels. All brackets were scanned in noncontact mode at T0 and T2. The coated brackets were scanned in contact mode at T1 to obtain better visualization of the nanoparticulate coating topography. At each time point, all groups were examined under identical instrument settings.

All roughness measurements were obtained over equivalent analysis areas at each time point. Roughness parameters were calculated within a region of interest selected at the center of the slot on each scan. For illustration, the post-friction images were captured over a larger field (30 × 30 µm) to show the full width of the wear tracks, whereas the pre-friction images are shown at 10 × 10 µm. Three measurements were obtained from separate points at the center of the slot of each bracket, and mean values of arithmetic average roughness (Ra), root mean square roughness (Rq), and maximum peak-to-valley height (Rz) were recorded in nanometers (Figures 4-5).

**Figure 4:**
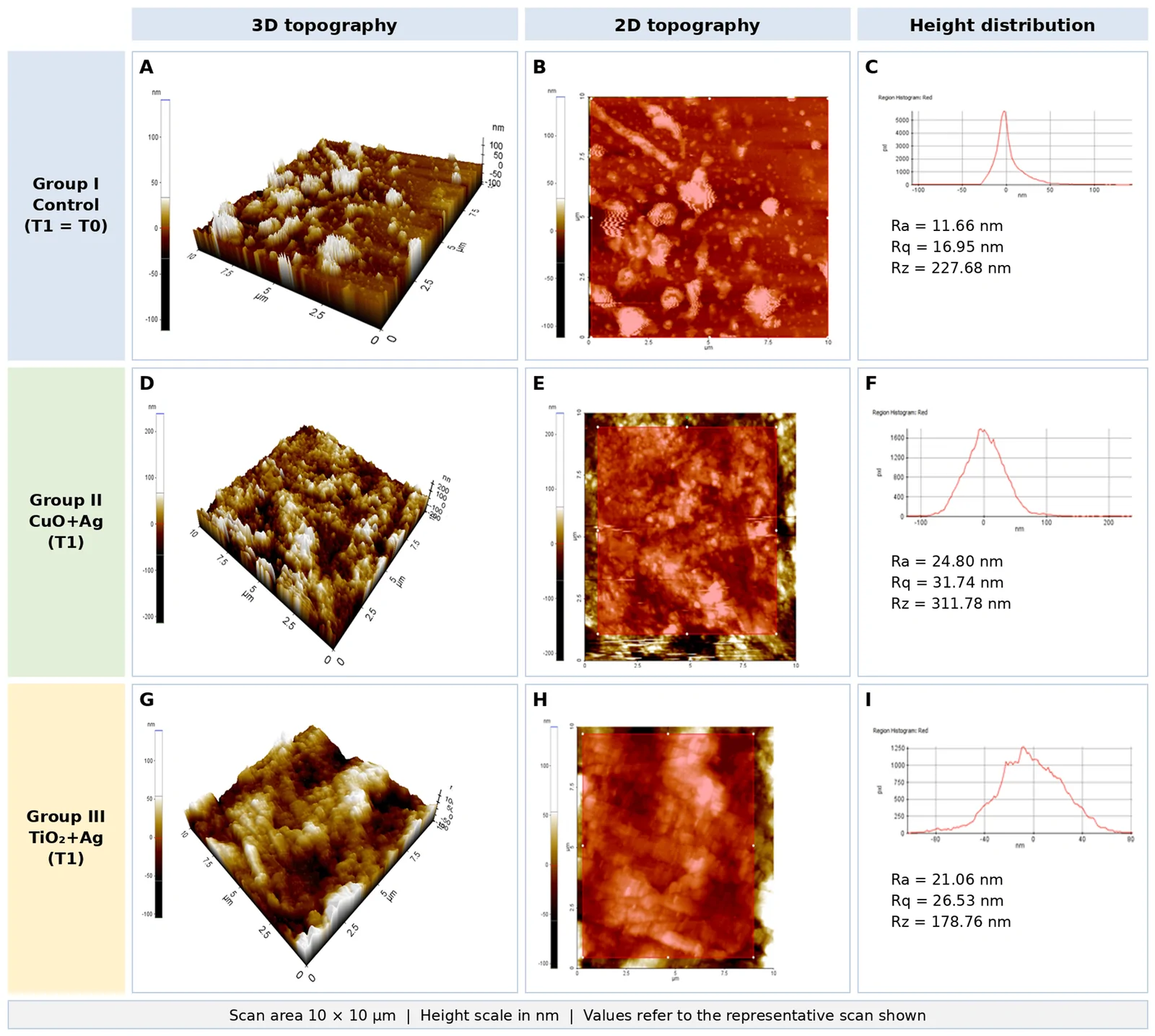
Representative atomic force microscopy analysis of the bracket slot before friction testing (T1) AFM: atomic force microscopy; Ra: arithmetic average roughness; Rq: root mean square roughness; Rz: maximum peak-to-valley height; CuO+Ag: silver-impregnated copper oxide; TiO₂+Ag: silver-impregnated titanium dioxide (A-C) Group I, uncoated control (T1 = T0); (D-F) Group II, CuO+Ag-coated brackets; and (G-I) Group III, TiO₂+Ag-coated brackets. The left column shows three-dimensional surface topography, the middle column shows two-dimensional topography, and the right column shows the corresponding height-distribution plots with Ra, Rq, and Rz values from the representative scans. Images are displayed over a 10 × 10 µm field, with height expressed in nm; roughness values were calculated within the analyzed region of interest. Values shown are from the representative scans

**Figure 5:**
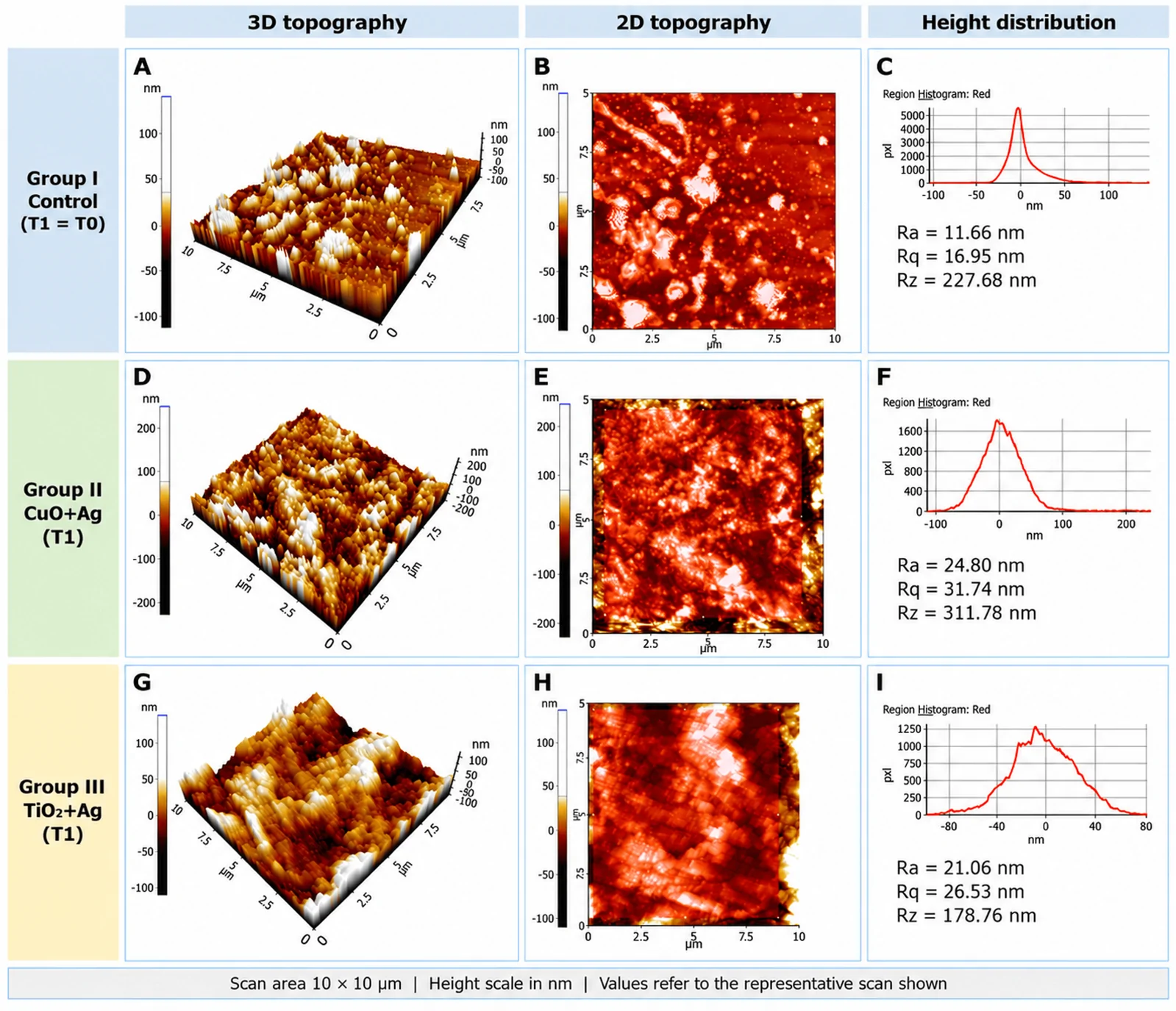
Representative atomic force microscopy analysis of the bracket slot after friction testing (T2) AFM: atomic force microscopy; Ra: arithmetic average roughness; Rq: root mean square roughness; Rz: maximum peak-to-valley height; CuO+Ag: silver-impregnated copper oxide; TiO₂+Ag: silver-impregnated titanium dioxide (A-C) Group I, uncoated control; (D-F) Group II, CuO+Ag-coated brackets; and (G-I) Group III, TiO₂+Ag-coated brackets. The left column shows three-dimensional surface topography, the middle column shows two-dimensional topography with the analyzed region indicated, and the right column shows the corresponding height-distribution plots with Ra, Rq, and Rz values from the representative scans. Images are displayed over a 30 × 30 µm field to show the wear tracks; roughness values were calculated within the indicated region of interest. Height scales are expressed in µm for panels A-F and nm for panels G-I. Values shown are from the representative scans

Blinding

Blinding was not performed because the coated and uncoated brackets were visually distinguishable by their surface appearance.

Statistical analysis

Data were entered into MS Excel (Microsoft Corporation, Redmond, Washington, United States) and analyzed using IBM SPSS Statistics for Windows, Version 29 (Released 2022; IBM Corp., Armonk, New York, United States). Continuous variables were summarized as mean ± standard deviation (SD). Normality was assessed using the Shapiro-Wilk test and homogeneity of variance using Levene’s test.

Intergroup comparisons at T0 and T1 were performed using ANOVA with the Tukey post hoc test when variances were homogeneous, and Welch ANOVA with the Games-Howell post hoc test when variances were unequal. Because baseline Ra deviated from normality in two groups, the Kruskal-Wallis test was also performed as a sensitivity analysis.

Changes from T0 to T1 and from T1 to T2 were assessed using paired t-tests, as the corresponding difference scores were normally distributed. Group I was not tested from T0 to T1 because its T1 values were identical to its T0 values by design. T1-to-T2 change scores were compared among groups using one-way ANOVA with the Tukey post hoc test, or Welch ANOVA with the Games-Howell post hoc test, as appropriate.

Static frictional resistance was compared among groups using one-way ANOVA followed by the Tukey post hoc test. Effect sizes were expressed as eta squared (η²), calculated from the sums of squares. Post hoc comparisons were reported as mean differences with 95% confidence intervals (CI) and the studentized range statistic (q). Statistical significance was set at p < 0.05.

## Results

All 30 brackets completed the experimental sequence and were included in the analysis. Descriptive statistics, normality testing, and homogeneity-of-variance testing for surface roughness and static frictional resistance are presented in Table 1.

**Table 1:** Descriptive statistics, normality, and homogeneity of variance for surface roughness and frictional resistance W: Shapiro-Wilk statistic; F: Levene statistic; df: degrees of freedom (between groups, within groups); SD: standard deviation; Ra: arithmetic average roughness; Rq: root mean square roughness; Rz: maximum peak-to-valley height; gf: gram-force; T0: as received; T1: after coating, before friction testing; T2: after friction testing; Group I: uncoated control; Group II: silver-impregnated copper oxide (CuO+Ag)-coated brackets; Group III: silver-impregnated titanium dioxide (TiO₂+Ag)-coated brackets Values are mean ± SD; n = 10 per group. Normality was assessed using the Shapiro-Wilk test and homogeneity of variance using Levene’s test. §p < 0.05: data not normally distributed. †p < 0.05: variances unequal; Welch ANOVA with the Games-Howell post hoc test was used. ‡Group I was not coated; its T1 values are identical to its T0 values. Difference scores for T0→T1 and T1→T2 were normally distributed in all groups (Shapiro-Wilk p > 0.05)

Time point	Parameter	Group (n = 10)	Mean ± SD	Normality assessment		Homogeneity of variance		
				Shapiro-Wilk W	p-value	Levene F	df	p-value
T0	Ra (nm)	I: Control	12.8 ± 1.8	0.801	0.015^§^	1.53	2, 27	0.234
		II: CuO+Ag	13.8 ± 2.1	0.790	0.011^§^			
		III: TiO₂+Ag	13.1 ± 1.1	0.922	0.376			
	Rq (nm)	I: Control	17.8 ± 1.9	0.966	0.848	0.67	2, 27	0.518
		II: CuO+Ag	18.1 ± 1.3	0.879	0.127			
		III: TiO₂+Ag	17.8 ± 1.7	0.969	0.882			
	Rz (nm)	I: Control	232.5 ± 6.4	0.924	0.389	0.73	2, 27	0.491
		II: CuO+Ag	234.8 ± 8.2	0.905	0.248			
		III: TiO₂+Ag	231.8 ± 8.6	0.943	0.592			
T1	Ra (nm)	I: Control	12.8 ± 1.8^‡^	0.801	0.015^§^	0.65	2, 27	0.532
		II: CuO+Ag	31.3 ± 3.1	0.986	0.989			
		III: TiO₂+Ag	24.5 ± 2.4	0.936	0.510			
	Rq (nm)	I: Control	17.8 ± 1.9^‡^	0.966	0.848	6.6	2, 27	0.005^†^
		II: CuO+Ag	36.1 ± 4.4	0.899	0.215			
		III: TiO₂+Ag	28.6 ± 2.4	0.876	0.116			
	Rz (nm)	I: Control	232.5 ± 6.4^‡^	0.924	0.389	6.58	2, 27	0.005^†^
		II: CuO+Ag	335.8 ± 17.8	0.964	0.830			
		III: TiO₂+Ag	182.7 ± 5.7	0.818	0.024^§^			
T2	Ra (nm)	I: Control	129.3 ± 4.5	0.951	0.680	1.27	2, 27	0.297
		II: CuO+Ag	91.3 ± 4.8	0.814	0.022^§^			
		III: TiO₂+Ag	66.5 ± 3.0	0.968	0.872			
	Rq (nm)	I: Control	168.2 ± 4.5	0.942	0.571	0.65	2, 27	0.528
		II: CuO+Ag	121.1 ± 2.8	0.974	0.923			
		III: TiO₂+Ag	86.2 ± 3.7	0.903	0.235			
	Rz (nm)	I: Control	853.1 ± 2.7	0.954	0.718	19.6	2, 27	<0.001^†^
		II: CuO+Ag	684.3 ± 27.0	0.944	0.597			
		III: TiO₂+Ag	751.3 ± 7.7	0.909	0.273			
Frictional resistance (measured between T1 and T2)	Static friction (gf)	I: Control	41.65 ± 4.77	0.899	0.213	0.14	2, 27	0.867
		II: CuO+Ag	36.31 ± 3.90	0.968	0.868			
		III: TiO₂+Ag	44.65 ± 4.53	0.965	0.839			

At T0, the three groups showed comparable surface roughness, with no significant differences in Ra (F(2, 27) = 0.93, p = 0.406, η² = 0.065), Rq (F(2, 27) = 0.11, p = 0.894, η² = 0.008), or Rz (F(2, 27) = 0.40, p = 0.674, η² = 0.029) (Table 2). Because Ra deviated from normality in Groups I and II, it was also analyzed with the Kruskal-Wallis test as a sensitivity analysis, which confirmed the absence of a baseline difference (H(2) = 0.86, p = 0.651).

**Table 2:** Baseline (T0) comparison of surface roughness among the study groups F: ANOVA statistic; df: degrees of freedom (between groups, within groups); η²: eta squared; T0: as-received condition before coating; Ra: arithmetic average roughness; Rq: root mean square roughness; Rz: maximum peak-to-valley height; SD: standard deviation; ANOVA: analysis of variant; Group I: uncoated control; Group II: silver-impregnated copper oxide (CuO+Ag)-coated brackets; Group III: silver-impregnated titanium dioxide (TiO₂+Ag)-coated brackets Values are mean ± SD; n = 10 per group. Intergroup comparisons were made by one-way ANOVA

Parameter (nm)	Group I: Control, mean ± SD	Group II: CuO+Ag, mean ± SD	Group III: TiO₂+Ag, mean ± SD	F	df	p-value	Effect size (η²)
Ra	12.8 ± 1.8	13.8 ± 2.1	13.1 ± 1.1	0.93	2, 27	0.406	0.065
Rq	17.8 ± 1.9	18.1 ± 1.3	17.8 ± 1.7	0.11	2, 27	0.894	0.008
Rz	232.5 ± 6.4	234.8 ± 8.2	231.8 ± 8.6	0.40	2, 27	0.674	0.029

At T1, both coatings significantly altered surface roughness. In Group II, Ra increased by 17.5 nm (+127%; t(9) = 15.65), Rq by 18.0 nm (+99%; t(9) = 13.42), and Rz by 101.0 nm (+43%; t(9) = 23.65) (all p < 0.001). In Group III, Ra increased by 11.5 nm (+88%; t(9) = 12.78) and Rq by 10.8 nm (+61%; t(9) = 10.82), whereas Rz decreased by 49.1 nm (-21%; t(9) = -18.23) (all p < 0.001) (Table 3). Significant intergroup differences were observed for Ra (F(2, 27) = 142.25, p < 0.001, η² = 0.913), Rq (Welch F(2, 16.69) = 105.62, p < 0.001, η² = 0.868), and Rz (Welch F(2, 16.48) = 409.97, p < 0.001, η² = 0.972). All pairwise comparisons were significant (q = 6.70-36.62, all p < 0.001) (Tables 3-4).

**Table 3:** Effect of nanoparticle coating on surface roughness: within-group change (T0 to T1) and between-group comparison at T1 ANOVA: analysis of variance; t (df): paired t-test statistic (degrees of freedom); F (df): ANOVA statistic (degrees of freedom, between groups and within groups); η²: eta squared, calculated from the sums of squares; CI: confidence interval; SD: standard deviation; Ra: arithmetic average roughness; Rq: root mean square roughness; Rz: maximum peak-to-valley height. Group I: uncoated control; Group II: silver-impregnated copper oxide (CuO+Ag)-coated brackets; Group III: silver-impregnated titanium dioxide (TiO₂+Ag)-coated brackets Values in nm; n = 10 per group. Within-group comparison (T0 vs T1): paired t-test. Between-group comparison at T1: ˣone-way ANOVA with the Tukey post hoc test; ʸWelch ANOVA with the Games-Howell post hoc test (unequal variances). Pairwise comparisons are presented in Table 4. *p < 0.05, statistically significant. ‡Group I was uncoated; its T1 values are identical to its T0 values, so no within-group test was applicable. Δ (T1 − T0): mean change from as received (T0) to post-coating (T1), with 95% CI; Δ (T1 − T0), % = (T1 − T0)/T0 × 100. Positive Δ values indicate an increase and negative values a decrease in roughness

Parameter (nm)	Group	T1, mean ± SD	Δ (T1-T0), mean (95% CI)	Δ (T1-T0), %	Intragroup comparison (T0 → T1), paired t-test		Intergroup comparison at T1		
					Within-group t(df)	Within-group p-value	Between-group F (df)	Between-group p-value	η²
Raˣ	I: Control‡	12.8 ± 1.8	0	0	-	-	142.25 (2, 27)	<0.001*	0.913
	II: CuO+Ag	31.3 ± 3.1	17.5 (15.0 to 20.0)	127	15.65 (9)	<0.001*			
	III: TiO₂+Ag	24.5 ± 2.4	11.5 (9.4 to 13.5)	88	12.78 (9)	<0.001*			
Rqʸ	I: Control‡	17.8 ± 1.9	0	0	-	-	105.62 (2, 16.69)	<0.001*	0.868
	II: CuO+Ag	36.1 ± 4.4	18.0 (15.0 to 21.0)	99	13.42 (9)	<0.001*			
	III: TiO₂+Ag	28.6 ± 2.4	10.8 (8.5 to 13.1)	61	10.82 (9)	<0.001*			
Rzʸ	I: Control‡	232.5 ± 6.4	0	0	-	-	409.97 (2, 16.48)	<0.001*	0.972
	II: CuO+Ag	335.8 ± 17.8	101.0 (91.4 to 110.7)	43	23.65 (9)	<0.001*			
	III: TiO₂+Ag	182.7 ± 5.7	-49.1 (-55.2 to -43.0)	-21	-18.23(9)	<0.001*			

**Table 4:** Post hoc pairwise comparison of surface roughness among the groups at T1 q: studentized range statistic; CI: confidence interval; Ra: arithmetic average roughness; Rq: root mean square roughness; Rz: maximum peak-to-valley height. Group I: uncoated control; Group II: silver-impregnated copper oxide (CuO+Ag)-coated brackets; Group III: silver-impregnated titanium dioxide (TiO₂+Ag)-coated brackets n = 10 per group; values in nm. Pairwise comparisons follow the between-group analysis in Table 3. Tukey post hoc test for Ra (equal variances) and Games–Howell post hoc test for Rq and Rz (unequal variances). p-values and 95% CIs are adjusted for multiple comparisons. *p < 0.05, statistically significant. Mean difference = first-listed group minus second-listed group; negative values indicate lower roughness in the first group. ‡Group I was uncoated; its T1 values were identical to its T0 values

Parameter (nm)	Post hoc test	Pairwise comparison	Mean difference (nm)	95% CI	q	Post hoc p-value
Ra	Tukey	I^‡^ vs II	-18.5	-21.3 to -15.8	23.58	<0.001*
		I^‡^ vs III	-11.7	-14.5 to -9.0	14.91	<0.001*
		II vs III	6.8	4.1 to 9.6	8.67	<0.001*
Rq	Games-Howell	I^‡^ vs II	-18.3	-22.3 to -14.3	17.17	<0.001*
		I^‡^ vs III	−10.8	-13.3 to -8.3	15.81	<0.001*
		II vs III	7.5	3.4 to 11.6	6.7	<0.001*
Rz	Games-Howell	I^‡^ vs II	-103.3	-119.4 to -87.2	24.42	<0.001*
		I^‡^ vs III	49.8	42.9 to 56.7	26.11	<0.001*
		II vs III	153.1	137.1 to 169.1	36.62	<0.001*

Ra and Rq were highest in Group II and lowest in Group I, whereas Rz was highest in Group II and lowest in Group III. Scanning electron microscopy showed a relatively smooth uncoated surface, dense irregular particulate deposits with CuO+Ag, and a more uniform particulate distribution with TiO₂+Ag (Figure 3). Atomic force microscopy demonstrated corresponding differences in surface topography before friction testing (Figure 4).

Static frictional resistance differed significantly among groups (F(2, 27) = 9.15, p < 0.001, η² = 0.404). Group II showed the lowest mean friction (36.31 ± 3.90 gf), followed by Group I (41.65 ± 4.77 gf) and Group III (44.65 ± 4.53 gf). Group II showed significantly lower friction than Group I (mean difference 5.34 gf; 95% CI 0.44 to 10.24; q = 3.82; p = 0.031) and Group III (mean difference: -8.34 gf; 95% CI: -13.24 to -3.44; q = 5.97; p < 0.001), whereas Group I and Group III did not differ significantly (mean difference: -3.00 gf; 95% CI: -7.90 to 1.90; q = 2.15; p = 0.298) (Table 5).

**Table 5:** Overall comparison (one-way ANOVA) and pairwise comparison (Tukey test) of static frictional resistance among the study groups during the friction run ANOVA: analysis of variance; Group I: uncoated control; Group II: silver-impregnated copper oxide (CuO+Ag)-coated brackets; Group III: silver-impregnated titanium dioxide (TiO₂+Ag)-coated brackets; df: degrees of freedom; SD: standard deviation; CI: confidence interval; η²: eta squared; q: studentized range statistic. Mean difference = first-listed group minus second-listed group; negative values indicate lower friction in the first group *p < 0.05, statistically significant.(gf: gram-force; 1 gf = 0.0098 N). One-way ANOVA was used for the overall comparison, followed by Tukey post hoc testing

Group	Static friction (gf), mean ± SD	Intergroup comparisons				Pairwise comparison				
		F-statistic	df	Overall p- value	Effect size (η²)	Groups	Mean difference (gf)	95% CI	q	Post hoc p-value
Group I: Control	41.65 ± 4.77	9.15	2, 27	<0.001*	0.404	I vs II	5.34	0.44 to 10.24	3.82	0.031*
Group II: CuO+Ag	36.31 ± 3.90					I vs III	-3.00	-7.90 to 1.90	2.15	0.298
Group III: TiO₂+Ag	44.65 ± 4.53					II vs III	-8.34	-13.24 to -3.44	5.97	<0.001*

Surface roughness increased significantly from T1 to T2 in all groups (paired t-test, t(9) = 33.48-304.27, all p < 0.001) (Table 6). The increases in Ra were 116.5 ± 4.5 nm in Group I, 60.0 ± 5.4 nm in Group II, and 42.0 ± 3.3 nm in Group III (F(2, 27) = 746.56, p < 0.001, η² = 0.982). Corresponding increases in Rq were 150.4 ± 4.1, 85.0 ± 6.1, and 57.6 ± 5.4 nm, respectively (F(2, 27) = 815.34, p < 0.001, η² = 0.984). For Rz, the increases were 620.6 ± 6.4, 348.5 ± 32.2, and 568.6 ± 8.6 nm, respectively (Welch F(2, 15.86) = 402.32, p < 0.001, η² = 0.976). All pairwise differences in change scores were significant (q = 12.65-55.58, all p < 0.001) (Table 7).

**Table 6:** Effect of friction testing on surface roughness: within-group change (T1 to T2) and between-group comparison of the change ANOVA: analysis of variance; t (df): paired t-test statistic (degrees of freedom); F (df): ANOVA statistic (degrees of freedom, between groups and within groups); η²: eta squared, calculated from the sums of squares; SD: standard deviation; Ra: arithmetic average roughness; Rq: root mean square roughness; Rz: maximum peak-to-valley height. Group I: uncoated control; Group II: silver-impregnated copper oxide (CuO+Ag)-coated brackets; Group III: silver-impregnated titanium dioxide (TiO₂+Ag)-coated brackets Values in nm; n = 10 per group. Within-group comparison (T1 vs T2): paired t-test. Between-group comparison of the change scores: ˣone-way ANOVA with the Tukey post hoc test; ʸWelch ANOVA with the Games-Howell post hoc test (unequal variances). Pairwise comparisons are presented in Table 7. *p < 0.05, statistically significant. ‡Group I was uncoated; its T1 values are identical to its T0 values. Δ (T2-T1): change from T1 to T2; positive values indicate an increase in roughness after friction testing

Parameter (nm)	Group	T2, mean ± SD	Δ (T2-T1), mean ± SD	Intra-group comparison (T1 to T2), paired t-test			Inter-group comparison (T1 to T2)		
				Within-group t (df)		Within-group p-value	Between-group F (df)	Between-group p-value	η²
Raˣ	I: Control‡	129.3 ± 4.5	116.5 ± 4.5	81.81 (9)	<0.001*		746.56 (2, 27)	<0.001*	0.982
	II: CuO+Ag	91.3 ± 4.8	60.0 ± 5.4	34.90 (9)	<0.001*				
	III: TiO₂+Ag	66.5 ± 3.0	42.0 ± 3.3	40.25 (9)	<0.001*				
Rqˣ	I: Control‡	168.2 ± 4.5	150.4 ± 4.1	115.58 (9)	<0.001*		815.34 (2, 27)	<0.001*	0.984
	II: CuO+Ag	121.1 ± 2.8	85.0 ± 6.1	44.12 (9)	<0.001*				
	III: TiO₂+Ag	86.2 ± 3.7	57.6 ± 5.4	33.48 (9)	<0.001*				
Rzʸ	I: Control‡	853.1 ± 2.7	620.6 ± 6.4	304.27 (9)	<0.001*		402.32 (2, 15.86)	<0.001*	0.976
	II: CuO+Ag	684.3 ± 27.0	348.5 ± 32.2	34.24 (9)	<0.001*				
	III: TiO₂+Ag	751.3 ± 7.7	568.6 ± 8.6	209.27 (9)	<0.001*				

**Table 7:** Post hoc pairwise comparison of the change in surface roughness after friction testing (T2-T1) among the groups Δ: change from T1 to T2; q: studentized range statistic; CI: confidence interval; Ra: arithmetic average roughness; Rq: root mean square roughness; Rz: maximum peak-to-valley height. Group I: uncoated control; Group II: silver-impregnated copper oxide (CuO+Ag)-coated brackets; Group III: silver-impregnated titanium dioxide (TiO₂+Ag)-coated brackets n = 10 per group; values in nm. Pairwise comparisons follow the between-group analysis in Table 6: Tukey post hoc test for ΔRa and ΔRq (equal variances) and Games-Howell post hoc test for ΔRz (unequal variances). p-values and 95% CIs are adjusted for multiple comparisons. *p < 0.05, statistically significant. Mean difference = first-listed group minus second-listed group; positive values indicate a greater increase in roughness in the first group. ‡Group I was uncoated; its T1 values are identical to its T0 values

Parameter (nm)	Post hoc test	Pairwise comparison				
		Groups	Mean difference (nm)	95% CI	q	Post hoc p-value
ΔRa	Tukey	I^‡^ vs II	56.5	51.5 to 61.5	39.71	<0.001*
		I^‡^ vs III	74.5	69.5 to 79.5	52.37	<0.001*
		II vs III	18.0	13.0 to 23.0	12.65	<0.001*
ΔRq	Tukey	I^‡^ vs II	65.4	59.6 to 71.3	39.17	<0.001*
		I^‡^ vs III	92.8	87.0 to 98.7	55.58	<0.001*
		II vs III	27.4	21.6 to 33.3	16.41	<0.001*
ΔRz	Games-Howell	I^‡^ vs II	272.1	243.5 to 300.7	37.07	<0.001*
		I^‡^ vs III	52.0	43.3 to 60.7	21.65	<0.001*
		II vs III	−220.1	−248.9 to −191.4	29.55	<0.001*

At T2, Group III showed the lowest Ra (66.5 ± 3.0 nm) and Rq (86.2 ± 3.7 nm), whereas Group II showed the lowest Rz (684.3 ± 27.0 nm). Group I showed the highest values for all three roughness parameters (Table 1, Table 6). Atomic force microscopy images after friction testing showed wear tracks in all groups (Figure 5).

## Discussion

The present study evaluated the effects of CuO+Ag and TiO₂+Ag nanoparticle coatings on the surface roughness and static frictional resistance of stainless steel orthodontic brackets. CuO+Ag produced the lowest frictional resistance, significantly lower than both the uncoated control and TiO₂+Ag groups, whereas TiO₂+Ag did not differ significantly from the control. Both coatings altered surface roughness at T1, and friction testing increased roughness in all groups. After friction testing, TiO₂+Ag showed the lowest Ra and Rq, whereas CuO+Ag showed the lowest Rz. The null hypothesis was therefore rejected.

Previous studies have reported inconsistent effects of nanoparticle coatings on orthodontic friction. Ghasemi et al. found increased friction with titanium oxide nanofilms, while Tawakal et al. reported increased friction with Ag- and Ag-chitosan-coated brackets [15,16]. Ameli et al. found no significant difference between CuO- or TiO₂-coated brackets and uncoated controls, whereas Ag-hydroxyapatite increased friction [17]. Similarly, Suresh et al. reported increased friction with TiO₂-coated archwires despite reduced surface roughness [18]. In contrast, other coatings, including aluminum oxide, titanium nitride, chromium nitride, zinc oxide, and chitosan, have reduced friction [23,24]. These differences suggest that frictional behavior depends on coating composition, deposition technique, coating integrity, and interaction with the opposing archwire surface [25].

In the present study, CuO+Ag significantly reduced friction compared with both TiO₂+Ag and the uncoated control. Ag-containing coatings alone have not consistently reduced friction [15,16], whereas more recent evidence suggests that Ag and TiO₂ nanocoatings can reduce friction when applied to orthodontic archwires [26]. The favorable behavior of CuO+Ag may therefore reflect the specific combination of coating materials and their interaction with the stainless steel bracket-wire interface. The present study, however, was not designed to determine the underlying tribological mechanism.

TiO₂+Ag showed the highest mean frictional resistance, although it was not significantly different from the uncoated control. Interestingly, it also showed relatively low Ra and Rq after friction testing. This confirms that surface roughness alone does not determine frictional behavior. Kusy and Whitley [12] and Choi et al. [13] similarly showed that the relationship between roughness and friction is complex. Adhesion, ploughing, binding, notching, bracket-wire geometry, and surface characteristics can all contribute to orthodontic friction.

The two coatings also produced different roughness profiles. CuO+Ag increased Ra, Rq, and Rz from T0 to T1, whereas TiO₂+Ag increased Ra and Rq but decreased Rz. This demonstrates the value of assessing more than one roughness parameter because Ra represents average surface deviations, Rq gives greater weighting to larger deviations, and Rz reflects peak-to-valley extremes. Previous studies have likewise shown that nanoparticle deposition can alter bracket surface topography, although the direction and magnitude of change vary with coating composition and technique [6,11,19].

Friction testing increased all roughness parameters, but the increases in Ra and Rq were smaller in both coated groups than in the uncoated control, while CuO+Ag showed the smallest increase in Rz. These findings indicate smaller friction-associated surface changes in the coated brackets. They should not, however, be interpreted as direct evidence of superior wear resistance because coating thickness, coating loss, wear volume, and coating retention were not quantitatively measured. Previous studies have similarly emphasized that coating integrity and mechanical stability are important determinants of performance [18,23,25].

Although antibacterial activity was not evaluated in this study, CuO+Ag and TiO₂+Ag were selected because of their reported antimicrobial potential. TiO₂-Ag-coated brackets have demonstrated activity against *S. mutans* and *P. gingivalis *[9], while CuO/Ag and Ag-Cu hybrid systems have shown favorable antibacterial effects [8,10,11]. However, current clinical evidence for nano-coated orthodontic materials remains limited [3,5,7]. An effective orthodontic coating should therefore combine antimicrobial activity with acceptable frictional behavior, mechanical stability, biocompatibility, and corrosion resistance.

CuO+Ag produced the most favorable frictional profile among the materials tested. Its lower static friction may be advantageous during sliding mechanics, but in vitro friction cannot be translated directly into the rate of clinical tooth movement because clinical mechanics are also influenced by binding, notching, bracket angulation, ligation, anchorage, biological response, and intraoral conditions.

All measured Ra values remained below the commonly cited 0.2-µm threshold associated with increased plaque retention [27], although the relevance of this threshold to nanocoated bracket slots requires clinical confirmation. CuO+Ag combined lower friction in the present study with antimicrobial properties reported previously [8,10,11], whereas TiO₂+Ag showed lower post-friction Ra and Rq without reducing friction compared with the control. This further demonstrates that surface smoothness alone should not determine coating selection.

This study was conducted under controlled in vitro conditions and cannot reproduce the full complexity of the oral environment, including variations in saliva, pH, temperature, mastication, biofilm, corrosion, and repeated loading. Only one bracket prescription, one 0.019 × 0.025-inch stainless steel archwire, elastomeric ligation, a 100-g load, 0° angulation, and static friction were evaluated. The standardized power-arm model allowed controlled comparison but represents only one bracket-wire configuration. Contact mode was used for the coated brackets at T1 to improve visualization of the coating, whereas non-contact mode was used at the other time points. Because the T1 values of the uncoated control were derived from its non-contact T0 scans, the between-group comparison at T1 may be partly influenced by the imaging mode. In addition, the slackening of the elastomeric module on the test bracket was standardized procedurally and verified by an unloaded trial run, but its ligation force was not quantitatively calibrated.

Coating thickness, coating retention, wear volume, nanoparticle release, corrosion resistance, and long-term durability were not assessed. Blinding was not possible because coated and uncoated brackets were visually distinguishable. Antibacterial effectiveness, cytotoxicity, ion release, and other biological outcomes were also not evaluated. Future studies should incorporate mechanical aging, quantitative coating-retention assessment, multispecies biofilm models, biological safety testing, and clinical evaluation.

## Conclusions

Within the limitations of this in vitro study, CuO+Ag and TiO₂+Ag nanocoatings altered the surface roughness of stainless steel orthodontic brackets. CuO+Ag increased Ra, Rq, and Rz, whereas TiO₂+Ag increased Ra and Rq but reduced Rz. CuO+Ag significantly reduced static frictional resistance compared with both the uncoated control and TiO₂+Ag, while TiO₂+Ag did not differ significantly from the control. Both coatings showed smaller friction-associated changes in surface roughness than uncoated brackets. As the coating with the lowest friction did not have the smoothest surface, surface roughness alone did not determine frictional resistance. CuO+Ag appears promising for further investigation, but its coating stability, antimicrobial activity, biocompatibility, and clinical performance require confirmation.
